# Applying a Positive (Organizational) Psychology Lens to the Study of Employee Green Behavior: A Systematic Review and Research Agenda

**DOI:** 10.3389/fpsyg.2022.840796

**Published:** 2022-04-26

**Authors:** Maria Christina Meyers, Demi Rutjens

**Affiliations:** Department of Human Resource Studies, Tilburg School of Social and Behavioral Sciences, Tilburg University, Tilburg, Netherlands

**Keywords:** positive organizational psychology, positive psychology, systematic review, employee green behavior, positive predictors

## Abstract

Employees can play a decisive role in combatting climate change by engaging in green behavior at work. Research on employee green behavior has recently gained traction, with research results pointing to the considerable influence of positive variables (e.g., personal values, positive affect) on employee green behavior. While such positive variables lie at the heart of the scholarly discipline positive organizational psychology, there is scant research at the intersection of positive organizational psychology and employee green behavior. The current manuscript aims to give impetus to such research. To this end, the manuscript presents a systematic review of the literature on positive predictors of employee green behavior and identified 94 articles that investigate such predictors. We explicitly map these investigated predictors onto a positive (organizational) psychology frame of reference. Subsequently, we use the findings of the review to identify gaps and outline concrete suggestions for future research at the intersection of positive organizational psychology and employee green behavior, addressing both theoretical and methodological suggestions.

## Introduction

Growing awareness of the impending threat of climate change has urged work- and organizational psychologists to start investigating employee green behavior (EGB), that is, workplace behavior that protects the environment against negative human influences (Ramus and Killmer, 2007; Daily et al., 2009; Boiral and Paillé, 2012; Ones and Dilchert, 2012; Paillé and Boiral, 2013). Employee green behavior includes actions such as recycling, using water sparingly, and making suggestions to improve organizational sustainability. In general, scholars differentiate between behavior that employees display in compliance with their official task descriptions (task-EGB; Ones and Dilchert, 2012), vs. voluntarily (voluntary EGB or organizational citizenship behavior for the environment; Daily et al., 2009; Paillé and Boiral, 2013). Task EGB can be measured in two ways. First, it may be measured by asking employees to what extent, in general, they fulfill their organizational responsibilities and duties in environmentally friendly ways (e.g., Bissing-Olson et al., 2013). Second, it may be measured by asking employees to what extent they perform specific pro-environmental behaviors, that is, to what extent they recycle, avoid waste, and conserve water, energy, or resources (e.g., Norton et al., 2017). Voluntary EGB involves pro-environmental behavior that is self-directed and that employees show spontaneously. It is commonly measured by a scale developed by Boiral and Paillé (2012) asking employees to indicate to what extent they voluntarily help coworkers perform their duties in a more environmentally-friendly manner, stay informed about and volunteer to participate in environmental initiatives of the organization, and put forward suggestions on how the organization can better protect the environment. Voluntary EGB can be operationalized as an overarching construct (e.g., Raineri and Paillé, 2016), or as one of its sub-dimensions, including eco-initiatives (proactively making suggestions, developing sustainable innovations), eco-helping (helping one's colleagues act in more sustainable ways), and eco-civic engagement (participation/engagement in environmental initiatives and activities) (Paillé and Boiral, 2013). No matter the exact definition or operationalization, scholars agree that green behavior of individual employees plays a major role in making organizations—as significant contributors to climate change—more sustainable (Ramus and Killmer, 2007; Ones and Dilchert, 2012; Norton et al., 2015; Boiral et al., 2018; Davis et al., 2020; Testa et al., 2020). In fact, research evidence corroborates that employee green behavior contributes to an organization's overall environmental performance (Paillé et al., 2014; Chen et al., 2015), suggesting that employees who act in environmentally friendly ways and inspire others to do so through their initiatives can make a marked difference in organizations.

Research on predictors of EGB has gained considerable traction in the past decade (Norton et al., 2015; Yuriev et al., 2018). Based on this research, we already know that predictors are situated at different levels. Predictors of EGB include, but are not limited to, perceptions of corporate social responsibility, green organizational climate, and green human resource management *at the organizational level* (e.g., Norton et al., 2017; De Roeck and Farooq, 2018; Zientara and Zamojska, 2018; Magill et al., 2020); green advocacy at the *workgroup- or team level* (Kim et al., 2017); moral reflectiveness, and pro-environmental values and attitudes at the *level of individual traits* (Boiral et al., 2015; Kim et al., 2017; Zientara and Zamojska, 2018); as well as affect (intrinsic), motivation, and commitment at the *level of individual states* (Bissing-Olson et al., 2013; Zientara and Zamojska, 2018; Paillé et al., 2019; Magill et al., 2020; Tian et al., 2020). Overall, this growing research seems to suggest that the most promising approach to increasing EGB is an inherently positive one—an approach that draws on positive norms and institutions (e.g., corporate social responsibility), positive traits (e.g., individual values), and positive states (e.g., positive affect, motivation) (Corral Verdugo, 2012).

Traditionally, positive institutions, positive individual traits, and positive states or experiences are the three central research domains (i.e., the three pillars) of positive psychology (Seligman and Csikszentmihalyi, 2000), and its sub-domain positive organizational psychology (Donaldson and Ko, 2010). Positive organizational psychology is “the scientific study of positive subjective experiences and traits in the workplace and positive organizations [*as a subset of positive institutions*], and its application to improve the effectiveness and quality of life in organizations” (Donaldson and Ko, 2010, p. 178). While positive psychology is dedicated to investigating overall human flourishing and optimal functioning (Gable and Haidt, 2005), positive organizational psychology is a narrower sub-domain investigating human flourishing and optimal functioning in the specific context of work and organizations (Donaldson and Dollwet, 2013). Prominent topics investigated in the positive organizational psychology literature are positive leadership, positive organizational development and change, and positive individual attributes of workers (Donaldson and Ko, 2010; Donaldson and Dollwet, 2013). Specifically, positive organizational psychology has generated a steady stream of research exploring positive, work-related predictors of employee health, wellbeing, relationships, and performance (Mills et al., 2013; Luthans and Youssef-Morgan, 2017; Bakker and van Woerkom, 2018). Commonly studied predictors include, but are not limited to, psychological capital (PsyCap; hope, self-efficacy, resilience, optimism) (Luthans and Youssef, 2007; Luthans and Youssef-Morgan, 2017), employee strengths use (Bakker and van Woerkom, 2018; Miglianico et al., 2020), positive leadership (Cameron et al., 2017), and passion at work (Vallerand et al., 2014). What remains under-explored among positive organizational psychologists, however, is how such positive, work-related predictors may relate to EGB.

While few articles have explicitly assessed predictors of EGB from a positive psychology perspective, we reason that the systematic application of positive psychology theory to the study of EGB can advance our understanding of who engages in EGB, under which circumstances and why they engage in EGB. Therefore, the present manuscript aims to inspire much-needed research at the intersection of positive organizational psychology and EGB. To this end, we first present a systematic review of the literature on predictors of EGB, in which we explicitly map these investigated predictors onto a positive psychology frame of reference. This frame of reference consists of the three main pillars of positive psychology, that is, positive subjective experiences, positive characteristics, and positive institutions (Seligman and Csikszentmihalyi, 2000). Overall, this review aims to uncover the current knowledge base on positive psychological constructs in relation to EGB. Second, we use the insights gained from the literature review to point out the gaps, and derive avenues for future research which draws on positive (organizational) psychology theory to advance our understanding of EGB.

Even though prior reviews on predictors of EGB exist (see Norton et al., 2015; Yuriev et al., 2018), the present review has a different focus and scope due to its unique emphasis on positive predictors. Specifically, it stands in direct contrast to the relatively recent review by Yuriev et al. (2018) focusing on barriers to EGB, that is, negative personal or organizational factors that impede employee engagement in green behavior. The current review will contribute to literature for years to come by providing a starting point and setting a research agenda for scholarly work at the intersection of positive psychology and sustainability. Specifically, it represents an important impulse to continue expanding the scope of positive organizational psychology research and practice to include our natural environment as one of its beneficiaries.

## Methods

### Literature Search

We conducted a systematic keyword search using the search engines PsycINFO and ABI/INFORM Global. Keywords included the following terms: *Employee Green Behavior, Employee Pro-Environmental Behavior, Organizational Citizenship Behavior for the Environment (OCBE)*, and *Employee Eco-Initiative*, as well as combinations of the terms *Organizational Citizenship Behavior, Employee*, and *Behavior*, with *Environment, Green*, and *Sustainable* (see Table 1 for the complete overview of search terms). We specifically searched for articles written in English, in peer-reviewed scholarly journals, covering adult populations. This resulted in a total of 1,031 identified articles on PsycINFO and 1,515 articles on ABI/Inform Global.

**Table 1:** Search strings used in the systematic literature review.

“Employee Green Behavior” OR “Employee Green Behavior”
“Employee Pro-Environmental Behavior” OR “Employee Pro-Environmental Behavior”
“Organizational Citizenship Behavior for the Environment” OR “Organizational Citizenship Behavior for the Environment” OR “Organizational Citizenship Behavior for the Environment” OR “Organizational Citizenship Behavior for the Environment”
“Organizational Citizenship Behavior” AND “Environment”
“Organizational Citizenship Behavior” AND “Environment”
“employee” AND “environment” AND “behavior”
“employee” AND “environment” AND “behavior”
“employee” AND “green” AND “behavior”
“employee” AND “green” AND “behavior”
“employee” AND “sustainable” AND “behavior”
“employee” AND “sustainable” AND “behavior”
“OCBE”
“employee eco-initiative”

We then scanned titles and abstracts of the identified articles to ascertain their relevance for this review. We excluded all articles that did not focus on (a) individual behavior, (b) behavior performed at work, and/or (c) behavior related to sustainability- or green goals. Furthermore, we excluded all articles that did not discuss antecedents of EGB. Finally, we excluded all double articles, resulting in a final total of 63 articles *via* PsycINFO and 69 articles *via* ABI/INFORM Global.

In the next step, we carefully read and coded these 132 articles in a schematic overview, extracting data on each article's study design, sample, sample size, measurement, studied predictors, and core theories, among others. We further narrowed down the selection of relevant articles by removing articles that did not report results of quantitative or mixed-method empirical studies. We also had to remove two articles that were retracted by their authors. Finally, and importantly, we removed articles that studied negative or neutral predictors of EGB (e.g., barriers to EGB). Note that we did not include the term positive psychology as keyword in our systematic literature search because the majority of articles on EGB cover constructs related to positive psychology without explicitly referring to them. In this step, we therefore carefully reviewed the predictors studied per article and selected only those studies that investigated constructs with clear links to the three pillars of positive psychology (i.e., positive subjective experiences, positive individual characteristics, and positive institutions) (Seligman and Csikszentmihalyi, 2000; Donaldson and Ko, 2010).

This resulted in a final sample of 94 articles (89 quantitative/5 mixed method) included in this review. Out of the 94 identified articles, 71 draw on cross-sectional data, 19 draw on cross-sectional, but time-lagged data, and four draw on longitudinal data. Out of the longitudinal studies, two are daily diary studies and two are (quasi-) experimental studies. To investigate EGB, most studies relied on self-report surveys, with a minority (*n* = 19) making use of other-ratings, mostly supervisor-ratings.

The samples included in these studies consisted of workers from all over the globe, with China (*k* = 13), Vietnam (*k* = 11), Pakistan (*k* = 8), and the UK (*k* = 6) as the four most represented countries. Overall, it appears that the empirical literature on employee green behavior is predominantly originating from Asian countries (*k* = 50), followed by European countries (*k* = 21), and Northern American countries (*k* = 12). Workers included in the samples worked in a variety of industries, but most studies were either conducted in the tourism and hospitality industry (k = 31), or with (convenience) samples of workers from different industries (k = 28). Sample sizes ranged from *N* = 47 to *N* = 1,230, with an average sample size of *N* = 367.

### Article Categorization

Following Seligman and Csikszentmihalyi (2000) and Donaldson and Ko (2010), we categorized predictors of EGB as belonging to the first pillar of *positive subjective experiences* when they pertained to positive feelings and other relatively fluctuating positive states, including happiness, wellbeing, flow, pleasure, hope, optimism, and positive emotions (Seligman and Csikszentmihalyi, 2000; Donaldson and Ko, 2010). We categorized variables under the second pillar of *positive individual characteristic* when they described character traits of individuals and other relatively stable (trait-like) attributes of a person, such as talents (knowledge, skills, or abilities), interests, creativity, wisdom, values or attitudes, character strengths, and courage (Seligman and Csikszentmihalyi, 2000; Donaldson and Ko, 2010).

The categorization of predictors under the third pillar of *positive institutions* was slightly more complex. In their original work on the different pillars of positive psychology, Seligman and Csikszentmihalyi (2000), described the third pillar as follows: “At the group level, it is about the civic virtues and the institutions that move individuals toward better citizenship: responsibility, nurturance, altruism, civility, moderation, tolerance, and work ethic” (p. 5). In line with this, van Rensburg and Rothmann (2020) define positive institutions as entities that:

Entertain a shared purpose and vision (of the moral goal of the institution), provide safety (protection against threats, danger and exploitation) and ensure fairness (equitable rules governing reward and punishment), humanity (care and concern) and dignity (treatment of all as individuals regardless of their position). (p. 2)

These definitions of positive institutions highlight that institutions are typically “shared” understandings or expectations, and manifest at the group level. Following these definitions, we categorized under *general positive institutions* different features of the organization or work group that move individuals within these organizations or groups toward better citizenship, often by entertaining a shared vision (think, for instance, of green human resource management), or by honoring common values such as safety, fairness, humanity, and dignity (think, for instance, of ethical leadership). At the same time, we found in the literature operationalizations of positive institutions as internalized by individuals. This is maybe not surprising given that institutions can be understood as formal rules or informal norms that govern the behavior of organizations, as well as individuals (Allard and Small, 2013). In the remainder of the manuscript, we therefore use the term *internalized positive institutions* to refer to the understood responsibilities or duties of individuals (think, for instance, of an individual's values or norms). Note that internalized institutions capture an individual's understanding of how a person should be or should behave, with reference to behavior that is considered good vs. bad, or right vs. wrong. In contrast to that, positive individual traits (Pillar 2) are simply about how a person is (free of judgment of whether this is right or wrong/good or bad).

## Results

For an overview of all articles that informed this review including the main predictors of EGB they investigate, please see the supplemental online material (Table A).

### Pillar 1: Positive Subjective Experiences

Forty-eight articles investigated one or more predictors (*k* = 58 predictors in total) to EGB that we considered positive subjective experiences based on their respective definition and operationalization. These were commitment (*k* = 17), fit (*k* = 11), motivation (*k* = 7), autonomy (*k* = 5), job satisfaction (*k* = 4), passion (*k* = 4), meaningfulness (*k* = 2), state-like self-efficacy (*k* = 2), trust (*k* = 2), wellbeing (*k* = 2), daily affect (*k* = 1), and environmental engagement (*k* = 1).

Overall, those articles widely supported the positive relationship between positive subjective experiences and employee green behavior. For instance, 16 of the 17 articles investigating commitment—be it to the organization (*k* = 8), the environment (*k* = 7), colleagues (*k* = 1), or goals (*k* = 1) (e.g., Paillé et al., 2016)—found support for a positive relationship with EGB. Only one study found mixed results, with a positive and significant correlation coefficient, but a non-significant regression coefficient for organizational commitment (Afsar et al., 2020). In addition, all 11 articles investigating individual perceptions of fit, or other forms of perceived value congruence between a person and their environment such as organizational identification, supported a positive relationship with EGB (e.g., Su and Swanson, 2019). Similarly, intrinsic motivation (*k* = 7), autonomy (*k* = 5), passion (*k* = 4), meaningfulness (*k* = 2), self-efficacy (*k* = 2), trust (*k* = 2), employee wellbeing (*k* = 2), and environmental engagement (*k* = 1) were all found to be positively related to EGB.

Only few of the investigated predictors showed less conclusive results. First, while two studies investigating job satisfaction found a positive relationship with EGB (Paillé and Boiral, 2013; Kim et al., 2019a), two other studies found either no, or a negative relationship (Paillé and Mejía-Morelos, 2014; Paillé et al., 2016). Second, a daily diary study on positive affect in relation to EGB (Bissing-Olson et al., 2013) indicated that daily unactivated positive affect (i.e., “contentment”) was positively related to EGB, whereas daily activated positive affect (i.e., “excitement”) was not.

### Pillar 2: Positive Individual Traits

Thirty-four articles investigated one or more predictors of EGB that were classified as positive individual traits or trait-like characteristics (*k* = 36 traits in total). These included a person's pro-environmental attitude (*k* = 23), green identity (*k* = 4), green competency or ability (*k* = 2), empathy (*k* = 2), internal locus of control (*k* = 2), future time perspective (*k* = 1), and generalized self-efficacy (*k* = 1).

Again, most articles supported the positive relationship between the investigated positive traits and EGB, in particular, for traits that directly relate to green or pro-environmental concerns. For instance, the most widely studied trait (-like) variable was pro-environmental attitude, referring to an individual's general awareness of environmental problems or belief in humanity's negative impact on the environment. It has been studied under several terms including workers' environmental concern (*k* = 9), -knowledge (*k* = 7), -awareness (*k* = 6), and -beliefs (*k* = 1). Overall, results of 21 out of 23 studies indicate a positive relationship between pro-environmental attitude and EGB (e.g., Zientara and Zamojska, 2018; Paillé et al., 2019). One study reported mixed results (Ahmed et al., 2020), meaning that the relationship between environmental knowledge and -concern and EGB was significant and positive, while the relationship between environmental awareness and EGB was not. One other study found a negative relationship between environmental beliefs and EGB (Chou, 2014).

Similarly, all four studies focusing on green identity, indicating that being environmentally friendly is integrated into a person's self-view (Luu, 2020a), found positive relationships between green identity and EGB. Note that one of the four studies only supported this relationship for two out of three dimensions of EGB (e.g., Luu, 2020b). Finally, green competence and—ability were also found unanimously to be positively related to EGB (Subramanian et al., 2016; Rayner and Morgan, 2018).

The results for more general positive traits such as empathy and internal locus of control were mixed. Empathy was found to be unrelated to EGB in two studies (Islam et al., 2019; Tian and Robertson, 2019). Internal locus of control (as part of an individual's core self-evaluation) was found to be positively related to EGB in one study (Robertson and Carleton, 2018), yet unrelated in another (Afsar et al., 2020). Results for generalized job-related self-efficacy pointed to a non-significant correlation between self-efficacy and EGB (Paillé et al., 2019). By contrast, future time perspective (having a more open-ended perception of the future) was positively related to EGB (Jiang et al., 2019).

### Pillar 3: Positive Institutions

In total, 76 articles were identified within the third pillar, out of which 7 covered internalized institutions, 60 covered general positive institutions, and 9 covered both types of institutions. This means that a total of 16 articles covered internalized institutions, while 69 articles covered general institutions.

#### Pillar 3A: Internalized Positive Institutions

The 16 identified articles under Pillar 3A each investigated one internalized positive institution (*k* = 16 internalized institutions in total). These were values (*k* = 7), morality (*k* = 4), norms (*k* = 4), and stages of consciousness (*k* = 1). With very few exceptions, these articles supported a positive relationship between internalized institutions and EGB. For instance, all articles focusing on either values (*k* = 7), broadly defined as “internalized social representations or moral beliefs that people appeal to as the ultimate rationale for their actions” (Oyserman, 2015, p. 37), or morality (*k* = 4), referring to the extent to which employees reflect on the virtuousness of their daily experiences and decisions (Kim et al., 2017; Afsar and Umrani, 2020), supported their respective positive relationships with EGB. Moreover, three out of four articles focusing on personal norms, that is, a person's sense of responsibility or obligation resulting from their values and personal code of ethics, supported a positive relationship with EGB. However, one article which conceptualized norms as “employees' recognition of the role of self-ethics and social responsibility in organizational effectiveness” (Zhao et al., 2021, p. 5), found no significant relationship.

#### Pillar 3B: General Positive Institutions

Reviewing the literature resulted in the identification of 69 articles that discuss one or several general positive institutions (*k* = 94 institutions in total), including support (*k* = 21), leadership (*k* = 18), green human resource management (*k* = 18) (green), climate (*k* = 15), corporate social responsibility (*k* = 10), green strategy (*k* = 8), corporate entrepreneurship (*k* = 2), organizational norms (*k* = 1), organizational justice (*k* = 1), and workplace spirituality (*k* = 1).

Again, the results reveal fairly consistent evidence for a positive relationship between positive institutions and EGB. Most notably, almost all tests investigating a form of perceived support, be it from the organization (*k* = 10), supervisors (*k* = 9), co-workers (*k* = 1), or top management (*k* = 1), corroborated a positive relationship with EGB (*k* = 18). Only two articles reported insignificant results (Erdogan et al., 2015; Afsar et al., 2016), while one article found positive results for one type of EGB, but non-significant for others (Manika et al., 2015). Next to general perceptions of support, various types of leadership were found to have a positive relationship with EGB, with 17 out of 18 tests yielding significant results. Among these are spiritual leadership (Afsar et al., 2016), responsible leadership (Afsar et al., 2020), ethical leadership (De Roeck and Farooq, 2018) (green), transformational leadership (Robertson and Carleton, 2018), servant leadership (Luu, 2019a), and empowering leadership (Jiang et al., 2019). Only one study found a non-significant relationship between green transformational leadership and EGB (Maziriri and Saurombe, 2018). Moreover, several variables that are indicative of green organizational policies, practices, and procedures were investigated, including green human resource management (GHRM), green climate, corporate social responsibility (CSR), and green strategy. Almost all studies supported the expected positive relationships between these variables and EGB. The only exceptions are two (out of *k* = 18) studies finding a non-significant relationship between GHRM and EGB (Pellegrini et al., 2018; Geiger et al., 2020), and one study (out of *k* = 15) finding a non-significant relationship between green climate and EGB (Norton et al., 2017).

### Investigation of Mediators and Moderators

While the above text focuses on direct relationships between various predictors and EGB, many of the articles included in our review develop and test complex research models, including mediators, moderators, or both. We were able to derive some general patterns in results regarding moderators and mediators.

First, variables pertaining to pillar 1 or positive subjective experiences are commonly studied as mediators in the included articles. For example, commitment (*k* = 13) and fit (*k* = 10) have consistently been studied as a mediator, serving as the mechanism that links general positive institutions (e.g., perceived organizational support, green HRM, or positive leadership) and EGB (see, e.g., Paillé and Morelos, 2017; Afsar and Umrani, 2020). Other variables that were found to mediate the link between general positive institutions and EGB are environmental engagement (Luu, 2019a), passion (Robertson and Barling, 2013; Afsar et al., 2016), self-efficacy (Testa et al., 2020), trust (Su and Swanson, 2019), and wellbeing (Ahmed et al., 2020).

Second, in contrast to pillar 1 variables, pillar 2 variables (positive individual traits) have often been included as moderators in the selected articles. For example, pro-environmental attitude was included as a moderator in several studies (*k* = 5). Among others, it was found to weaken the relationship between daily activated positive affect and EGB (Bissing-Olson et al., 2013). Another example is green identity, which was found to strengthen the relationship between both leadership (Wang et al., 2018; Luu, 2020b) and green communication as part of HRM (Luu, 2020a), and EGB. Other positive individual trait variables that have been investigated as moderators were empathy (Tian and Robertson, 2019), internal locus of control (Robertson and Carleton, 2018; Afsar et al., 2020), future time perspective (Jiang et al., 2019), and general self-efficacy (Paillé et al., 2019).

Third, internalized positive institutions (Pillar 3a) have been studied as both moderators and mediators in the included articles. On the one hand, values have been studied as moderator in two studies, yielding mixed results. The first study found that green values strengthen the relationship between green climate and voluntary EGB, but do not influence the link between green climate and task EGB (Dumont et al., 2017). By contrast, another study found that strong green values might weaken the relationship between Corporate Environmental Responsibility (CER) and EGB (Ruepert et al., 2017). On the other hand, morality has been found to mediate the relationship between both personal characteristics such as conscientiousness (Kim et al., 2017) and EGB, and organizational initiatives such as CSR (Afsar and Umrani, 2020) and HRM (Luu, 2020a) and EGB (*k* = 3). Because there are comparatively fewer studies that include variables pertaining to this pillar, we cannot derive any definite patterns based on these results yet.

Fourth, general positive institutions (Pillar 3b) have often been studied as predictors in the included articles, exerting their influence on EGB via, for instance, positive subjective experiences (see Pillar 1). However, several selected articles also pointed out potential synergies between different general positive institutions in moderation analyses. For instance, both ethical (De Roeck and Farooq, 2018) and servant leadership (Luu, 2017, 2019b) were found to strengthen the positive relationship between CSR and EGB (*k* = 3). Moreover, green climate was found to strengthen the positive relationships between, respectively, green HRM (Luu, 2018), leadership (Khan et al., 2019), and training (Pham et al., 2018), and EGB (*k* = 5). In stark contrast to these synergetic effects stand the results of studies investigating green climate as a moderator of the relationships between positive individual traits or internalized institutions and EGB. In these studies, green climate was found to weaken the link between both personal green norms (Chou, 2014) and attitudes (Tian et al., 2020) and EGB (*k* = 3). Finally, studies have also recognized that one positive institution may influence EGB *via* another positive institution in mediation analyses. Most notably, green climate has been supported as a mediator (*k* = 8) in the relationships between organization-level variables such as leadership (e.g., Kim et al., 2017; Khan et al., 2019) and green policies (Norton et al., 2014), and EGB.

## Discussion

This systematic literature review aimed to provide an overview of the current research on employee green behavior in relation to the three pillars of positive psychology (i.e., positive subjective experiences, positive individual traits, and positive institutions). Even though there is only little explicit emphasis on positive (organizational) psychology in the EGB literature, we found and reviewed 94 empirical articles that focused on predictors of EGB, which, in the broadest sense, can be considered “positive” concepts falling under the three pillars of positive psychology. Overall, this review points out that exploring positive avenues toward stimulating EGB is a worthwhile endeavor because results of a large majority of studies revealed positive relationships between variables pertaining to all three pillars of positive psychology and EGB.

### Current State of Theoretical Developments

Various theories may explain these positive relationships. In the reviewed articles, the most commonly referenced theory is the Theory of Planned Behavior (TPB; Ajzen, 1991) (*n* = 17), followed by Social Identity Theory (Tajfel and Turner, 1986) (*n* = 13), and Social Exchange Theory (SET; Blau, 1964) (*n* = 12). Other theories that were drawn on repeatedly are the Value Beliefs Norms Theory (VBN; Stern, 2000) (*n* = 7) and Self-Determination Theory (SDT; Ryan and Deci, 2000; Deci and Ryan, 2008) (*n* = 6). Finally, due to its special place in positive psychology, we also refer to the broaden-and-build theory of positive emotions (Fredrickson, 1998, 2001) mentioned in *n* = 2 of the reviewed articles. Below, we outline how these theories can be used to explain the main results of our review per pillar of positive psychology.

First, our literature review unveiled consistent positive relationships between positive subjective experiences (Pillar 1; e.g., commitment, motivation, job satisfaction, positive affect) and EGB. These relationships have been commonly explained by SDT (Ryan and Deci, 2000; Deci and Ryan, 2008), proposing that autonomous motivation (including intrinsic motivation) does not only stimulate individual wellbeing and performance, but also individual perseverance and consistency in the display of behaviors. The theory may therefore explain the positive relationships between (green) intrinsic motivation and EGB, in particular, concerning *voluntary* (extra-role) EGB (see, e.g., Tian et al., 2020). Furthermore, two studies draw on the broaden-and-build theory of positive emotions (Fredrickson, 1998, 2001) to explain this relationship. This theory suggests that subjective experiences of positive emotions broaden an individual's momentary thought-action repertoire, leading to the exploration of new and creative action alternatives. Literature suggests that green behaviors are examples of such new and creative actions (Bissing-Olson et al., 2013; Kim et al., 2019a). However, Bissing-Olson et al. (2013) also note that these behaviors can only be considered new and creative for people who are normally not very concerned about the environment. People who are concerned about it, will habitually display green behaviors at work. This contingency on environmental concern may explain why the expected positive relationship between satisfaction or affect and EGB was not corroborated in a minority of studies (Bissing-Olson et al., 2013; Paillé and Mejía-Morelos, 2014; Paillé et al., 2016).

Second, our review corroborated positive relationships between positive individual characteristics (Pillar 2; e.g., pro-environmental attitude, green identity, locus of control, future time perspective) and EGB. These relationships have often been explained by two related theories. The first is TPB (Ajzen, 1991), postulating that behavior—including green behavior—is strongly determined by a person's behavioral intentions. Behavioral intentions, in turn, result from attitudes toward the behavior in question, subjective norms, and perceived behavioral control. In relation to the second pillar, this theory has been used to explain the relationships between positive attitudes toward green behavior and EGB (e.g., Lamm et al., 2013; Wang, 2016), as well as between self-efficacy (as an indicator of behavioral control) and EGB (Testa et al., 2020). The second related theory is the VBN Theory (Stern, 2000), proposing that values influence pro-environmental behavior *via* pro-environmental beliefs and personal norms. In line with this, VBN theory has been used to explain that employees are more likely to engage in EGB when they have pro-environmental attitudes or beliefs (see, e.g., Verplanken et al., 2008; Rezapouraghdam et al., 2018). These theories can also be used to explain why more general positive traits such as empathy are not always found to be positively related to EGB. These more general traits are not specifically linked to attitudes or norms regarding the environment, and would thus not necessarily translate into intentions regarding green behavior (Ajzen, 1991).

Third, the same two theories (TPB and VBN) have been used to explain the relationship between a person's internalized positive institutions (Pillar 3a; e.g., values, morality, norms, and stages of consciousness) and EGB. These theories argue that internalized institutions, such as values and norms, are strong predictors of displayed behaviors. Within the reviewed articles, TPB and VBN have been used conjointly to explain the relationship between, respectively, work ethic (Peng and Lee, 2019) and norms (Chou, 2014), and EGB. In addition, TPB has been used to explain the link between values and EGB (Boiral et al., 2015). Interestingly, the use of the same theories under pillar 2 and 3a may indicate that the distinction between positive individual traits and internalized positive institutions is a subtle one, and that the positive characteristics that matter for EGB may be closely tied to internalized institutions. However, we still see an important distinction between the two in that positive individual traits capture how a person is (*how would you describe that person?*) without passing judgment, whereas internalized positive institutions capture beliefs or convictions about how a person should be or behave (*how should the person behave?*), which entails a normative judgment of right and wrong, or good and bad.

Moreover, our review corroborated positive relationships between general positive institutions (Pillar 3b; e.g., support, positive leadership, GHRM, green climate, CSR, and green strategy) and EGB, which have commonly been explained by social exchange theory (SET; Blau, 1964) in the reviewed literature. This theory suggests that social exchange relationships depend on a careful balance of giving and taking among the involved parties. One party's investment in the relationship thus compels the other party to reciprocate with a similar investment (Gouldner, 1960). Social exchange relationships can also form between organizations and their employees (Rhoades and Eisenberger, 2002), where organizational investments in employees, which go beyond investments that are determined by the employment contract (e.g., extra HR initiatives or support), compel employees to return the favor. The social exchange reasoning suggests that employees, who perceive initiatives such as green HRM, green climate, or CSR as an extra investment in them, will engage in green behaviors to fulfill their perceived social obligations toward the organization (see, e.g., Gkorezis, 2015; Bhatnagar and Aggarwal, 2020). However, note that the lack of written agreements in social exchange relationships may also explain why some studies did not find the expected relationship between general positive institutions and EGB (e.g., Norton et al., 2017; Maziriri and Saurombe, 2018). While investments by the organization or leader will compel employees to reciprocate, the employees will still have to make an informed guess on the type of reciprocation that is most valued by the other party. If employees do not perceive strong signals that green behavior is highly valued by the other party, or if they perceive other signals to be stronger (e.g., that cost savings are valued), they may reciprocate with other behaviors than green behaviors. At the same time, the relationship between general positive institutions and EGB may also be explained by economic exchange relationships between organizations and their employees. Employees will engage in green behaviors if their contractual or economic exchange relationship with the organization mandates it and some organization clearly steer employees toward green behavior through their green policies, procedures, or practices (e.g., through rewards for green behavior as part of GHRM).

Finally, to explain the mediating roles of positive subjective experiences (most notably, commitment and fit) in the relationship between general positive institutions and EGB, the reviewed articles often refer to social identity theory (Tajfel and Turner, 1986). This theory suggests that individuals' self-concepts are highly contingent on their social group memberships. To bolster their self-concepts, individuals strive to be members of social groups with a high public standing. Pursuing green or sustainable initiatives (e.g., CSR, green HRM, green climate, green strategy) improves the public standing of organizations. Consequently, these initiatives entice employees to solidify their membership of these organizations, by strongly attaching themselves to the organizations (e.g., through commitment) or by integrating organizational values into their self-view (e.g., through organizational identification or fit) (Kim et al., 2019b; Su and Swanson, 2019). Subsequently, employees will be eager to display behavior (e.g., green behavior) that further benefits the standing of the organization they are committed to or identify with. By extension, this will also benefit their self-concept (Su and Swanson, 2019).

### Where Do We Go From Here? Future Positive Psychological Research on EGB

**Figure 1:**
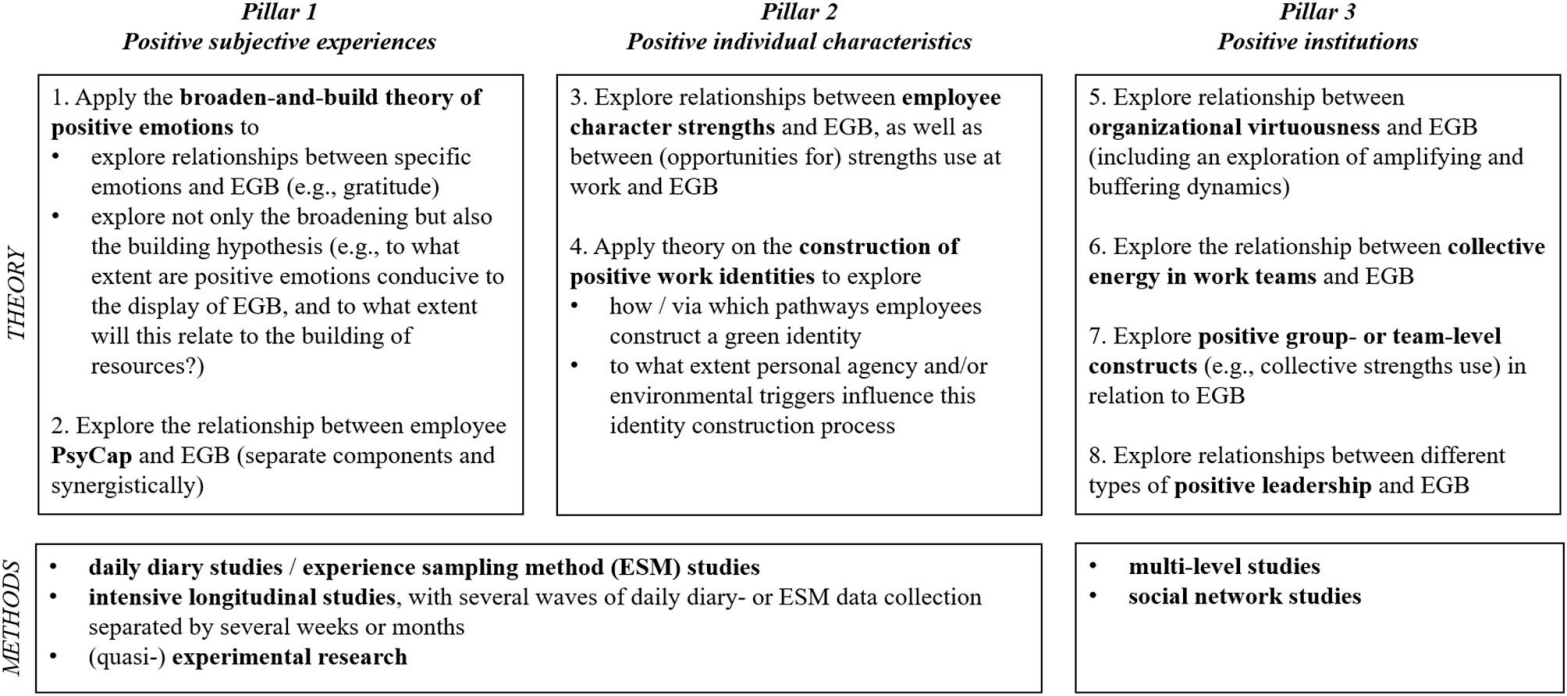
Overview of suggested avenues for future research on employee green behavior per pillar of positive psychology.

Our literature review suggests that pursuing positive avenues toward stimulating EGB is worthwhile because the review uncovered consistent positive relationships between variables relating to the three pillars of positive psychology and EGB. At the same time, the current state of theoretical development indicates that there is little explicit emphasis on positive psychology theorizing to date. We thus foresee many relevant avenues for future research at the intersection of positive organizational psychology and EGB (for an overview, see Figure 1).

#### Pillar 1—Positive Subjective Experiences

We suggest that future research on the relationship between positive subjective experiences and EGB focuses on applying the broaden-and-build theory of positive emotions (Fredrickson, 2001), which remains underexplored to date (for exceptions, see Bissing-Olson et al., 2013; Kim et al., 2019a). The finding that activated (e.g., joy) and un-activated (e.g., contentment) positive emotions display differential relationships with EGB (Bissing-Olson et al., 2013) calls for more research into the relationships between specific types of positive emotions (e.g., joy, interest, contentment, pride, love, gratitude) and EGB. We reason that gratitude may be of particular interest: As a positive emotion that is other-directed (i.e., we are grateful to someone, or for something) (Emmons and McCullough, 2003), it may also relate to behavior that is other-directed, such as EGB. Zooming in on specific emotions allows researchers to test the *broadening* hypothesis of the theory in more nuanced ways. In addition, researchers still have to test the *building* hypothesis of the theory (Fredrickson, 1998, 2001). This hypothesis suggests that EGB as a *broadened* thought-action repertoire may contribute to *building* personal resources (e.g., social connections, empathy, mindfulness, environmental knowledge) over time, which, in turn, facilitate future positive experiences (i.e., gain spirals).

We also think that future research on positive psychological capital (PsyCap; Luthans and Youssef, 2007; Luthans and Youssef-Morgan, 2017) in relation to EGB is worth pursuing. While prior research has already focused on self-efficacy as one of the PsyCap components (Testa et al., 2020), there is little research on the other three components (hope, optimism, resilience) in relation to EGB. We deem assessing all four PsyCap components worthwhile because there is strong research evidence for positive effects of all four components on employee attitudes, behavior, and performance (Luthans and Youssef-Morgan, 2017). In addition, the components may have synergistic effects, which are worth exploring.

#### Pillar 2—Positive Individual Traits

Building on the supported positive relationships between positive individual traits and EGB, we advise positive organizational psychologists to explore the link between employee character strengths (i.e., positively valued traits) and EGB. By definition, character strengths are rooted in virtues (Peterson and Seligman, 2004; McGrath and Brown, 2020), which are considered human characteristics that bring benefits to both the person who possesses the virtue as well as to others, in their surroundings (Foot, 1997; McGrath and Brown, 2020). Due to these roots, character strengths are also closely tied to the display of virtuous behavior (e.g., someone with kindness as their strength is likely to display acts of kindness), which makes them likely antecedents of EGB. While empirical studies on the relationship between character strengths and green behavior in the context of work are hitherto lacking, initial research evidence has already supported links between character strengths and green behavior of tourists (Warren and Coghlan, 2016). In addition, character strengths have been linked to green or pro-environmental self-efficacy in a convenience sample of US Americans (Moeller and Stahlmann, 2019). We therefore deem it very promising to explore how different employee character strengths relate to EGB, including the mechanisms and boundary conditions that affect these relationships in the work context. Relatedly, it seems promising to explore how strengths use (Miglianico et al., 2020) or the applicability of strengths at work (Harzer and Ruch, 2013) relate to EGB. This is particularly relevant because using or applying strengths at work fosters experiences of positive emotions and intrinsic motivation (Miglianico et al., 2020), which are relevant situational antecedents of EGB (Norton et al., 2015).

Furthermore, prior research supports a positive relationship between a person's green identity and EGB. We suggest that green identities can be considered *positive identities* because they appear infused with virtuous qualities (e.g., humanity, caring, self-control) (Dutton et al., 2010). Consequently, it becomes useful to integrate literature on EGB with theory on the construction of positive work identities (Dutton et al., 2010). This theory may inspire scholars to explore how or *via* which pathways employees construct a green identity, and to what extent personal agency and/or environmental triggers influence this identity construction process. Profound insights into how a green work identity is built may help to derive successful organizational interventions to enhance EGB.

#### Pillar 3—Positive Institutions

We foresee four particularly fruitful avenues for future research on positive institutions and EGB. First, future research may link EGB to organizational virtuousness, which is about the pursuit of human flourishing and excellence, striving to do what is morally good and right, and an unconditional devotion to creating social value that transcends the instrumental interests of any specific actor (Bright et al., 2006). Organizational virtuousness can trigger two specific types of dynamics: amplifying dynamics or gain spirals where current virtuous actions (e.g., doing good, displaying compassion, being honest about and taking responsibility for mistakes) inspire future virtuous actions (tonic virtuousness), and buffering dynamics where the impact of negative events such as downsizing or mergers is cushioned by virtuous actions (e.g., leaders protecting the wellbeing of their followers; colleagues who stimulate each other to use their full potential despite the trying times) that are shown in response to it (Bright et al., 2006). In the context of research on EGB, it appears particularly interesting to study amplifying dynamics to explore the patterns and developments in the display of EGB throughout an organization. Second and relatedly, EGB may be linked to theory on the collective energy in work teams (Cross et al., 2003; Cole et al., 2012). Collective energy is an emergent phenomenon, rooted in individual energetic states that fan out and amplify through interactions, exchanges, shared exposure to events, and contagion processes among team members (Cole et al., 2012). Collective energy is functional in stimulating desired behaviors because it triggers affective, cognitive, and behavioral tendencies that propel teams toward higher functioning. It remains to be studied whether collective energy can also propel teams and their individual members toward displaying more EGB. Third, it may be interesting to study other positive group- or team-level constructs in relation to EGB. Think, for instance, of “dream teams” that benefit from several positive inputs (e.g., team diversity, team attachment) and processes (e.g., optimism, supportive leadership) to achieve optimal team functioning (Richardson and West, 2010). Specifically, the emergent research on either strengths use (van Woerkom et al., 2020) or strengths-based roles (Gander et al., 2020) in work teams seems relevant here, given that strengths research points to links with both positive functioning and virtuousness (McGrath and Brown, 2020; Miglianico et al., 2020). Finally, we encourage future research on positive leadership (authentic, respectful, servant, spiritual, and/or strengths-based leadership) in relation to EGB. Positive leaders inspire others to excel by exuding the moral values and ethical standards of an organization (Mills et al., 2013; Cameron et al., 2017). As such, positive leaders are role models of moral and ethical behavior, potentially including green behavior, which may inspire others to follow suit.

### Methodological Directions

To pursue the above avenues for future research, we suggest that scholars move away from the cross-sectional designs that have dominated EGB research (Norton et al., 2015) toward other, more advanced, research designs including longitudinal and multi-level designs. Specifically, daily diary studies such as in Bissing-Olson et al. (2013) or experience sampling method (ESM) studies (Larson and Csikszentmihalyi, 2014) are needed to investigate links between momentary experiences (e.g., emotions) and EGB. Moreover, intensive longitudinal studies, with several waves of daily diary- or ESM data collection separated by several weeks or months (for an example, see Quintus et al., 2020), allow researchers to link day-to-day or momentary experiences to longer term developments. These studies would be uniquely suitable to test not only the broadening-, but also the building hypothesis of positive emotions (Fredrickson, 2001), as well as processes of green identity development (Dutton et al., 2010). Furthermore, scholars may use (quasi-) experimental research to test the effects of targeted, positive workplace interventions on EGB, *via* constructs such as PsyCap (Luthans and Youssef-Morgan, 2017), strengths use (Miglianico et al., 2020), or gratitude (Emmons and McCullough, 2003). It would be interesting to consider boundary conditions of effects because workplace interventions may not always work for all employees under all circumstances (Unsworth et al., 2013; Nielsen and Miraglia, 2017). To conduct research on general positive institutions (e.g., organizational virtuousness) and EGB, researchers should conduct multi-level studies accounting for the fact that employees are nested in work teams or organizations (Norton et al., 2015). In addition, to capture the complex social dynamics that may contribute to EGB (e.g., amplifying dynamics, spread of energy, being inspired by leaders), we require dyadic, or even better, social network studies (Scott, 1988). The latter would allow to explore how and where positive movements originate, as well as which people are involved in which role (as energizers, followers, gatekeepers, etc.).

### Limitations

While this review opens up novel research directions at the intersection of positive organizational psychology and EGB, it is also subject to some limitations. First, we only searched for articles using overarching key terms (e.g., EGB) and not specific, single green behaviors (e.g., recycling, using public transport). In that, we followed Ones and Dilchert (2012) assuming that single indicators are too narrowly focused to be useful in building a scientific understanding of employee green behaviors. Second, we only considered articles that explored green behavior in the work context, but not other contexts, most notably the context of a person's home. Research has shown that green behavior are not always transferable across contexts, as each context entails specific incentives, obstacles, and opportunities (Lo et al., 2012). For instance, the wish or need to cut down own expenses may act as a strong incentive to conserve resources (e.g., water, electricity, gas) at home, that is not present at work. This calls for context-specific examinations. Third, the finding that almost all included studies reported positive relationships between the investigated variables and EGB needs to be interpreted with some caution. Although the open science movement has led to reductions in the file drawer problem (the tendency to only publish signifcant results; Rosenthal, 1979), the relationship between positive constructs and EGB may still be overestimated based on the included studies. Relatedly, there still are considerable shortcomings in the scientific rigor in several of the studies included in this review (e.g., cross-sectional designs; ambiguous definition or operationalization of key variables) that necessitate us to interpret the findings of this review with some caution.

## Conclusion

The findings of this review suggest that positive organizational psychology may have a crucial role to play in furthering our understanding of predictors of employee green behavior. With climate change as “the defining issue of our time” (UN, 2021) and organizations as a major contributor to climate change (Ones and Dilchert, 2012; Norton et al., 2015), we call to the community of scholars to apply positive psychological theorizing in the pursuit of more sustainable and green actions of both employees and organizations.

## Data Availability Statement

The original contributions presented in the study are included in the article/Supplementary Materials, further inquiries can be directed to the corresponding author.

## Author Contributions

MM contributed to the conception, design of the study, and wrote the current draft of the manuscript. MM and DR conducted the systematic review. DR organized the database and wrote sections of the manuscript. Both authors approved the submitted draft.

## Conflict of Interest

The authors declare that the research was conducted in the absence of any commercial or financial relationships that could be construed as a potential conflict of interest.

## Publisher's Note

All claims expressed in this article are solely those of the authors and do not necessarily represent those of their affiliated organizations, or those of the publisher, the editors and the reviewers. Any product that may be evaluated in this article, or claim that may be made by its manufacturer, is not guaranteed or endorsed by the publisher.
