# Supplementary material for: Applying a Positive (Organizational) Psychology Lens to the Study of Employee Green Behavior: A Systematic Review and Research Agenda
Source: Front Psychol. 2022 Apr 26;13:840796. doi: 10.3389/fpsyg.2022.840796 (PMC9087848; doi:10.3389/fpsyg.2022.840796)
Supplement: Supplementary file 1 [file Table_1.DOCX]

Supplementary Material

Applying a Positive (Organizational) Psychology Lens to the Study of Employee Green Behavior: A Systematic Review and Research Agenda

Table A

*Overview of Identified Positive Predictors of Employee Green Behavior Mapped onto the Three Pillars of Positive Psychology*

| **Pillar** | **Positive predictor** | **#** | **References** |
| --- | --- | --- | --- |
| ***Pillar 1: Positive Subjective Experiences*** | | | |
| 1 | commitment | 17 | (Afsar et al., 2020; Afsar & Umrani, 2020; Davis et al., 2020; Kim et al., 2019; Lamm et al., 2013; Luu, 2019b; Paillé & Boiral, 2013; Paillé et al., 2016; Paillé, Mejía Morelos, et al., 2019; Paillé & Morelos, 2017; Pellegrini et al., 2018; Raineri & Paillé, 2016; Safari et al., 2018; Temminck et al., 2015; Wang, 2016; Zientara & Zamojska, 2018) |
| 1 | fit | 11 | (Chaudhary, 2020; Cheema et al., 2020; De Roeck & Farooq, 2018; Islam et al., 2019; Luu, 2019a; Nejati et al., 2019; Su & Swanson, 2019; Tian & Robertson, 2019; Tosti-Kharas et al., 2017; Wang et al., 2018; Zhao et al., 2019) |
| 1 | motivation | 7 | (Afsar et al., 2016; Davis et al., 2020; Kim et al., 2016; Magill et al., 2020; Rayner & Morgan, 2018; Stritch & Christensen, 2016; Tian et al., 2020) |
| 1 | autonomy | 5 | (Boiral et al., 2015; Cop et al., 2020; Jiang et al., 2019; Lamm et al., 2015; Paillé & Morelos, 2017) |
| 1 | job satisfaction | 4 | (A. Kim et al., 2019; Paillé & Boiral, 2013; Paillé & Mejía-Morelos, 2014; Paillé et al., 2016) |
| 1 | passion | 4 | (Afsar et al., 2016; Maziriri & Saurombe, 2018; Robertson & Barling, 2013; Saifulina & Carballo-Penela, 2017) |
| 1 | meaningfulness | 2 | (Bhatnagar & Aggarwal, 2020; Luu, 2020a) |
| 1 | state-like self-efficacy | 2 | (Kim et al., 2016; Testa et al., 2020) |
| 1 | trust | 2 | (Paillé, Raineri, et al., 2019; Su & Swanson, 2019) |
| 1 | well-being | 2 | (Ahmed et al., 2020; Su & Swanson, 2019) |
| 1 | daily affect | 1 | (Bissing-Olson et al., 2013) |
| 1 | environmental engagement | 1 | (Luu, 2019a) |
| ***Pillar 2 - Positive Individual Traits*** | | | |
| 2 | pro-environmental attitude | 23 | (Afsar et al., 2016; Ahmed et al., 2020; Bissing-Olson et al., 2013; Chan et al., 2017; Chou, 2014; Kim et al., 2016; Kura, 2016; Lamm et al., 2013; Manika et al., 2015; Norton et al., 2014; Okumus et al., 2019; Paillé et al., 2014; Rayner & Morgan, 2018; Rezapouraghdam et al., 2018; Saeed et al., 2019; Safari et al., 2018; Tariq et al., 2020; Temminck et al., 2015; Teng et al., 2014; Tian et al., 2020; Tudor et al., 2007, 2008; Verplanken et al., 2008) |
| 2 | green identity | 4 | (Luu, 2020a, 2020c; Wang et al., 2018; Wang, 2016) |
| 2 | green competency or ability | 2 | (Rayner & Morgan, 2018; Subramanian et al., 2016) |
| 2 | empathy | 2 | (Islam et al., 2019; Tian & Robertson, 2019) |
| 2 | internal locus of control | 2 | (Afsar et al., 2020; Robertson & Carleton, 2018) |
| 2 | future time perspective | 1 | (Jiang et al., 2019) |
| 2 | generalized self-efficacy | 1 | (Paillé, Raineri, et al., 2019) |
| ***Pillar 3A – Internalized Positive Institutions*** | | | |
| 3A | values | 7 | (Boiral et al., 2015; Chaudhary, 2020; Dumont et al., 2017; Peng & Lee, 2019; Ruepert et al., 2017; Wahab, 2017; Zientara & Zamojska, 2018) |
| 3A | morality | 4 | (Afsar & Umrani, 2020; Kim et al., 2017; Luu, 2020a; Wang, 2016) |
| 3A | norms | 4 | (Chou, 2014; Han et al., 2019; Tosti-Kharas et al., 2017; Zhao et al., 2019) |
| 3A | stages of consciousness | 1 | (Boiral et al., 2018) |
| ***Pillar 3B - General Positive Institutions*** | | | |
| 3B | support | 21^[[1]](#footnote-1)^ | (Afsar et al., 2016; Bhatnagar & Aggarwal, 2020; Erdogan et al., 2015; Gkorezis, 2015; Han et al., 2019; Lamm et al., 2015; Lamm et al., 2013; Luu, 2019a; Manika et al., 2015; Paillé & Boiral, 2013; Paillé et al., 2013; Paillé et al., 2016; Paillé, Mejía Morelos, et al., 2019; Paillé & Morelos, 2017; Paillé, Raineri, et al., 2019; Pellegrini et al., 2018; Raineri & Paillé, 2016; Tariq et al., 2020; Testa et al., 2020) |
| 3B | leadership | 18 | (Afsar et al., 2016; Afsar et al., 2020; De Roeck & Farooq, 2018; Han et al., 2019; Jiang et al., 2019; Khan et al., 2019; Luu, 2017a, 2019a, 2019b, 2019c, 2019d, 2020b, 2020c; Maziriri & Saurombe, 2018; Nejati et al., 2019; Robertson & Barling, 2013; Robertson & Carleton, 2018; Wang, 2016) |
| 3B | green human resource management (HRM) | 18 | (Chaudhary, 2019, 2020; Cop et al., 2020; Dumont et al., 2017; Emilisia & Lunarindiah, 2020; Fahim et al., 2019; Geiger et al., 2020; Y. J. Kim et al., 2019; Law et al., 2017; Luu, 2018, 2019d, 2020b; Pellegrini et al., 2018; Pham et al., 2018; Pham et al., 2019; Rayner & Morgan, 2018; Saeed et al., 2019; Zhao et al., 2019) |
| 3B | climate | 15 | (Afsar & Umrani, 2020; Chou, 2014; Dumont et al., 2017; Khan et al., 2019; Kim et al., 2017; Luu, 2018, 2019c; Magill et al., 2020; Norton et al., 2014; Norton et al., 2017; Pham et al., 2018; Robertson & Carleton, 2018; Saeed et al., 2019; Tian et al., 2020; Zientara & Zamojska, 2018) |
| 3B | corporate social responsibility | 10 | (Afsar et al., 2020; Ahmed et al., 2020; Bohdanowicz et al., 2011; De Roeck & Farooq, 2018; Islam et al., 2019; Luu, 2017a; Luu, 2017b; Ruepert et al., 2017; Su & Swanson, 2019; Tian & Robertson, 2019) |
| 3B | green strategy | 8 | (Chen et al., 2015; García-Mira et al., 2017; Norton et al., 2014; Norton et al., 2017; Paillé et al., 2013; Paillé et al., 2014; Pellegrini et al., 2018; Raineri & Paillé, 2016) |
| 3B | corporate entrepreneurship | 1 | (Luu, 2017b) |
| 3B | norms | 1 | (Pandur & Albulescu, 2016) |
| 3B | organizational justice | 1 | (Luu, 2019b) |
| 3B | workplace spirituality | 1 | (Rezapouraghdam et al., 2018) |

*Note.* # Number of times a predictor has been investigated in the identified articles**;** most often, this number is equal to the number of articles that have investigated a predictor; for the only exception, see Footnote 1.

**References**

Afsar, B., Badir, Y., & Kiani, U. S. (2016). Linking spiritual leadership and employee pro-environmental behavior: The influence of workplace spirituality, intrinsic motivation, and environmental passion. *Journal of Environmental Psychology*, *45*, 79-88. h[ttps://doi.org/10.1016/j.jenvp.2015.11.011](ttps://doi.org/10.1016/j.jenvp.2015.11.011%20)

Afsar, B., Maqsoom, A., Shahjehan, A., Afridi, S. A., Nawaz, A., & Fazliani, H. (2020). Responsible leadership and employee's proenvironmental behavior: The role of organizational commitment, geen shared vision, and internal environmental locus of control. *Corporate Social Responsibility and Environmental Management*, *27*, 297-312. h[ttps://doi.org/10.1002/csr.1806](ttps://doi.org/10.1002/csr.1806%20)

Afsar, B., & Umrani, W. A. (2020). Corporate social responsibility and pro-environmental behavior at workplace: The role of moral reflectiveness, coworker advocacy, and environmental commitment. *Corporate Social Responsibility and Environmental Management*, *27*(1), 109-125. h[ttps://doi.org/https://doi.org/10.1002/csr.1777](ttps://doi.org/https://doi.org/10.1002/csr.1777%20)

Ahmed, M., Zehou, S., Raza, S. A., Qureshi, M. A., & Yousufi, S. Q. (2020). Impact of CSR and environmental triggers on employee green behavior: The mediating effect of employee well‐being. *Corporate Social Responsibility and Environmental Management*, *27*(5), 2225-2239. h[ttps://doi.org/10.1002/csr.1960](ttps://doi.org/10.1002/csr.1960%20)

Bhatnagar, J., & Aggarwal, P. (2020). Meaningful work as a mediator between perceived organizational support for environment and employee eco-initiatives, psychological capital and alienation. *Employee Relations: The International Journal*, *42*(6), 1487-1511. h[ttps://doi.org/10.1108/er-04-2019-0187](ttps://doi.org/10.1108/er-04-2019-0187%20)

Bissing-Olson, M. J., Iyer, A., Fielding, K. S., & Zacher, H. (2013). Relationships between daily affect and pro-environmental behavior at work: The moderating role of pro-environmental attitude. *Journal of Organizational Behavior*, *34*(2), 156-175. h[ttps://doi.org/https://doi.org/10.1002/job.1788](ttps://doi.org/https://doi.org/10.1002/job.1788%20)

Bohdanowicz, P., Zientara, P., & Novotna, E. (2011). International hotel chains and environmental protection: an analysis of Hilton's we care! programme (Europe, 2006–2008). *Journal of Sustainable Tourism*, *19*(7), 797-816.

Boiral, O., Raineri, N., & Talbot, D. (2018). Managers’ citizenship behaviors for the environment: A developmental perspective. *Journal of Business Ethics*, *149*(2), 395-409. h[ttps://doi.org/10.1007/s10551-016-3098-6](ttps://doi.org/10.1007/s10551-016-3098-6%20)

Boiral, O., Talbot, D., & Paillé, P. (2015). Leading by example: A model of organizational citizenship behavior for the environment. *Business Strategy and the Environment*, *24*(6), 532-550. h[ttps://doi.org/https://doi.org/10.1002/bse.1835](ttps://doi.org/https://doi.org/10.1002/bse.1835%20)

Chan, E. S. W., Hon, A. H. Y., Okumus, F., & Chan, W. (2017). An Empirical Study of Environmental Practices and Employee Ecological Behavior in the Hotel Industry. *Journal of Hospitality & Tourism Research*, *41*(5), 585-608. h[ttps://doi.org/10.1177/1096348014550873](ttps://doi.org/10.1177/1096348014550873%20)

Chaudhary, R. (2019). Green human resource management in Indian automobile industry. *Journal of Global Responsibility*, *10*(2), 161-175. h[ttps://doi.org/10.1108/jgr-12-2018-0084](ttps://doi.org/10.1108/jgr-12-2018-0084%20)

Chaudhary, R. (2020). Green human resource management and employee green behavior: An empirical analysis. *Corporate Social Responsibility and Environmental Management*, *27*(2), 630-641. h[ttps://doi.org/https://doi.org/10.1002/csr.1827](ttps://doi.org/https://doi.org/10.1002/csr.1827%20)

Cheema, S., Afsar, B., & Javed, F. (2020). Employees' corporate social responsibility perceptions and organizational citizenship behaviors for the environment: The mediating roles of organizational identification and environmental orientation fit. *Corporate Social Responsibility and Environmental Management*, *27*(1), 9-21. h[ttps://doi.org/10.1002/csr.1769](ttps://doi.org/10.1002/csr.1769%20)

Chen, Y., Tang, G., Jin, J., Li, J., & Paillé, P. (2015). Linking market orientation and environmental performance: The influence of environmental strategy, employee's environmental involvement, and environmental product quality [Article]. *Journal of Business Ethics*, *127*(2), 479-500. h[ttps://doi.org/10.1007/s10551-014-2059-1](ttps://doi.org/10.1007/s10551-014-2059-1%20)

Chou, C.-J. (2014). Hotels' environmental policies and employee personal environmental beliefs: Interactions and outcomes. *Tourism Management*, *40*, 436-446. h[ttps://doi.org/https://doi.org/10.1016/j.tourman.2013.08.001](ttps://doi.org/https://doi.org/10.1016/j.tourman.2013.08.001%20)

Cop, S., Alola, U. V., & Alola, A. A. (2020). Perceived behavioral control as a mediator of hotels' green training, environmental commitment, and organizational citizenship behavior: A sustainable environmental practice. *Business Strategy and the Environment*, *29*(8), 3495-3508. h[ttps://doi.org/https://doi.org/10.1002/bse.2592](ttps://doi.org/https://doi.org/10.1002/bse.2592%20)

Davis, M. C., Unsworth, K. L., Russell, S. V., & Galvan, J. J. (2020). Can green behaviors really be increased for all employees? Trade‐offs for 'deep greens' in a goal‐oriented green human resource management intervention. *Business Strategy and the Environment*, *29*(2), 335-346. h[ttps://doi.org/10.1002/bse.2367](ttps://doi.org/10.1002/bse.2367%20)

De Roeck, K., & Farooq, O. (2018). Corporate social responsibility and ethical leadership: Investigating their interactive effect on employees’ socially responsible behaviors. *Journal of Business Ethics*, *151*(4), 923-939. h[ttps://doi.org/10.1007/s10551-017-3656-6](ttps://doi.org/10.1007/s10551-017-3656-6%20)

Dumont, J., Shen, J., & Deng, X. (2017). Effects of green HRM practices on employee workplace green behavior: The role of psychological green climate and employee green values. *Human Resource Management*, *56*(4), 613-627. h[ttps://doi.org/10.1002/hrm.21792](ttps://doi.org/10.1002/hrm.21792%20)

Emilisia, N., & Lunarindiah, G. (2020). Consequences of Green Human Resource Management: Perspective of Professional Event Organizer Employees in Jakarta. *Review of Integrative Business and Economics Research*, *9*, 361-372.

Erdogan, B., Bauer, T. N., & Taylor, S. (2015). Management commitment to the ecological environment and employees: Implications for employee attitudes and citizenship behaviors. *Human Relations*, *68*(11), 1669-1691. h[ttps://doi.org/10.1177/0018726714565723](ttps://doi.org/10.1177/0018726714565723%20)

Fahim, F., Khan, N. R., Ahmad, A., & Ali, A. (2019). Green human resource management and firm's environmental performance: Mediating role of employee commitment, green involvement and eco-friendly Behavior. *Paradigms*, *13*(2), 18-25. h[ttps://doi.org/http://dx.doi.org/10.24312/1969130203](ttps://doi.org/http://dx.doi.org/10.24312/1969130203%20)

García-Mira, R., Dumitru, A., Alonso-Betanzos, A., Sánchez-Maroño, N., Fontenla-Romero, Ó., Craig, T., & Polhill, J. G. (2017). Testing scenarios to achieve workplace sustainability goals using backcasting and agent-based modeling. *Environment and Behavior*, *49*(9), 1007-1037. h[ttps://doi.org/10.1177/0013916516673869](ttps://doi.org/10.1177/0013916516673869%20)

Geiger, S. M., Fischer, D., Schrader, U., & Grossman, P. (2020). Meditating for the planet: Effects of a mindfulness-based intervention on sustainable consumption behaviors. *Environment and Behavior*, *52*(9), 1012-1042. h[ttps://doi.org/10.1177/0013916519880897](ttps://doi.org/10.1177/0013916519880897%20)

Gkorezis, P. (2015). Supervisor support and pro-environmental behavior: the mediating role of LMX. *Management Decision*, *53*(5), 1045-1060. h[ttps://doi.org/10.1108/md-06-2014-0370](ttps://doi.org/10.1108/md-06-2014-0370%20)

Han, Z., Wang, Q., & Yan, X. (2019). How responsible leadership predicts organizational citizenship behavior for the environment in China. *Leadership & Organization Development Journal*, *40*(3), 305-318. h[ttps://doi.org/10.1108/LODJ-07-2018-0256](ttps://doi.org/10.1108/LODJ-07-2018-0256%20)

Islam, T., Ali, G., & Asad, H. (2019). Environmental CSR and pro-environmental behaviors to reduce environmental dilapidation. *Management Research Review*, *42*(3), 332-351. h[ttps://doi.org/10.1108/mrr-12-2017-0408](ttps://doi.org/10.1108/mrr-12-2017-0408%20)

Jiang, M., Wang, H., & Li, M. (2019). Linking empowering leadership and organizational citizenship behavior toward environment: The role of psychological ownership and future time perspective. *Frontiers in psychology*, *10*. h[ttps://doi.org/10.3389/fpsyg.2019.02612](ttps://doi.org/10.3389/fpsyg.2019.02612%20)

Khan, M. A. S., Jianguo, D., Ali, M., Saleem, S., & Usman, M. (2019). Interrelations between ethical leadership, green psychological climate, and organizational environmental citizenship behavior: A moderated mediation model. *Frontiers in psychology*, *10*. h[ttps://doi.org/10.3389/fpsyg.2019.01977](ttps://doi.org/10.3389/fpsyg.2019.01977%20)

Kim, A., Kim, Y., & Han, K. (2019). A cross level investigation on the linkage between job satisfaction and voluntary workplace green behavior. *Journal of Business Ethics*, *159*(4), 1199-1214. h[ttps://doi.org/10.1007/s10551-018-3776-7](ttps://doi.org/10.1007/s10551-018-3776-7%20)

Kim, A., Kim, Y., Han, K., Jackson, S. E., & Ployhart, R. E. (2017). Multilevel influences on voluntary workplace green behavior: individual differences, leader behavior, and coworker advocacy. *Journal of Management*, *43*(5), 1335-1358. h[ttps://doi.org/10.1177/0149206314547386](ttps://doi.org/10.1177/0149206314547386%20)

Kim, S.-H., Kim, M., Han, H.-S., & Holland, S. (2016). The determinants of hospitality employees’ pro-environmental behaviors: The moderating role of generational differences. *International Journal of Hospitality Management*, *52*, 56-67. h[ttps://doi.org/https://doi.org/10.1016/j.ijhm.2015.09.013](ttps://doi.org/https://doi.org/10.1016/j.ijhm.2015.09.013%20)

Kim, Y. J., Kim, W. G., Choi, H.-M., & Phetvaroon, K. (2019). The effect of green human resource management on hotel employees’ eco-friendly behavior and environmental performance. *International Journal of Hospitality Management*, *76*, 83-93. h[ttps://doi.org/10.1016/j.ijhm.2018.04.007](ttps://doi.org/10.1016/j.ijhm.2018.04.007%20)

Kura, K. M. (2016). Linking Environmentally Specific Transformational Leadership and Environmental Concern to Green Behavior at Work. *Global Business Review*, *17*(3_suppl), 1S-14S. h[ttps://doi.org/10.1177/0972150916631069](ttps://doi.org/10.1177/0972150916631069%20)

Lamm, E., Tosti-Kharas, J., & King, C. E. (2015). Empowering employee sustainability: Perceived organizational support toward the environment. *Journal of Business Ethics*, *128*(1), 207-220. h[ttps://doi.org/10.1007/s10551-014-2093-z](ttps://doi.org/10.1007/s10551-014-2093-z%20)

Lamm, E., Tosti-Kharas, J., & Williams, E. G. (2013). Read this article, but don’t print it: Organizational citizenship behavior toward the environment. *Group & Organization Management*, *38*(2), 163-197. h[ttps://doi.org/10.1177/1059601112475210](ttps://doi.org/10.1177/1059601112475210%20)

Law, M. M. S., Hills, P., & Hau, B. C. H. (2017). Engaging employees in sustainable development—A case study of environmental education and awareness training in Hong Kong. *Business Strategy and the Environment*, *26*(1), 84-97. h[ttps://doi.org/10.1002/bse.1903](ttps://doi.org/10.1002/bse.1903%20)

Luu, T. T. (2017a). Activating tourists' citizenship behavior for the environment: the roles of CSR and frontline employees' citizenship behavior for the environment. *Journal of Sustainable Tourism*, *26*(7), 1178-1203. h[ttps://doi.org/10.1080/09669582.2017.1330337](ttps://doi.org/10.1080/09669582.2017.1330337%20)

Luu, T. T. (2017b). CSR and organizational citizenship behavior for the environment in hotel industry. *International Journal of Contemporary Hospitality Management*, *29*(11), 2867-2900. h[ttps://doi.org/10.1108/ijchm-02-2016-0080](ttps://doi.org/10.1108/ijchm-02-2016-0080%20)

Luu, T. T. (2018). Employees’ green recovery performance: the roles of green HR practices and serving culture. *Journal of Sustainable Tourism*, *26*(8), 1308-1324. h[ttps://doi.org/10.1080/09669582.2018.1443113](ttps://doi.org/10.1080/09669582.2018.1443113%20)

Luu, T. T. (2019a). Building employees’ organizational citizenship behavior for the environment: The role of environmentally-specific servant leadership and a moderated mediation mechanism. *International Journal of Contemporary Hospitality Management*, *31*(1), 406-426. h[ttps://doi.org/10.1108/IJCHM-07-2017-0425](ttps://doi.org/10.1108/IJCHM-07-2017-0425%20)

Luu, T. T. (2019b). Catalyzing employee OCBE in tour companies: Charismatic leadership, organizational justice, and pro-environmental behaviors. *Journal of Hospitality & Tourism Research*, *43*(5), 682-711. h[ttps://doi.org/10.1177/1096348018817582](ttps://doi.org/10.1177/1096348018817582%20)

Luu, T. T. (2019c). Effects of environmentally-specific servant leadership on green performance via green climate and green crafting. *Asia Pacific Journal of Management*, 1-29. h[ttps://doi.org/http://dx.doi.org/10.1007/s10490-019-09687-9](ttps://doi.org/http://dx.doi.org/10.1007/s10490-019-09687-9%20)

Luu, T. T. (2019d). Green human resource practices and organizational citizenship behavior for the environment: the roles of collective green crafting and environmentally specific servant leadership. *Journal of Sustainable Tourism*, *27*(8), 1167-1196. h[ttps://doi.org/10.1080/09669582.2019.1601731](ttps://doi.org/10.1080/09669582.2019.1601731%20)

Luu, T. T. (2020a). Reducing food waste behavior among hospitality employees through communication: dual mediation paths. *International Journal of Contemporary Hospitality Management*, *32*(5), 1881-1904. h[ttps://doi.org/10.1108/ijchm-09-2019-0779](ttps://doi.org/10.1108/ijchm-09-2019-0779%20)

Luu, T. T. (2020b). Integrating green strategy and green human resource practices to trigger individual and organizational green performance: the role of environmentally-specific servant leadership. *Journal of Sustainable Tourism*, *28*(8), 1193-1222. h[ttps://doi.org/10.1080/09669582.2020.1729165](ttps://doi.org/10.1080/09669582.2020.1729165%20)

Luu, T. T. (2020c). Environmentally-specific servant leadership and green creativity among tourism employees: dual mediation paths. *Journal of Sustainable Tourism*, *28*(1), 86-109. h[ttps://doi.org/10.1080/09669582.2019.1675674](ttps://doi.org/10.1080/09669582.2019.1675674%20)

Magill, M. S., Yost, P. R., Chighizola, B., & Stark, A. (2020). Organizational climate for climate sustainability. *Consulting Psychology Journal: Practice and Research*, *72*(3), 198-222. h[ttps://doi.org/10.1037/cpb0000163](ttps://doi.org/10.1037/cpb0000163%20)

Manika, D., Wells, V. K., Gregory-Smith, D., & Gentry, M. (2015). The Impact of Individual Attitudinal and Organisational Variables on Workplace Environmentally Friendly Behaviors. *Journal of Business Ethics*, *126*(4), 663-684. h[ttps://doi.org/10.1007/s10551-013-1978-6](ttps://doi.org/10.1007/s10551-013-1978-6%20)

Maziriri, E. T., & Saurombe, M. D. (2018). Antecedents towards employees’ harmonious habitation of the environment and workplace environment-friendly Behavior: a case of Johannesburg employees within small and medium enterprises (SMES). . *Journal of Business and Retail Management Research*, *13*(1).

Nejati, M., Salamzadeh, Y., & Loke, C. K. (2019). Can ethical leaders drive employees’ CSR engagement? *Social Responsibility Journal*, *16*(5), 655-669. h[ttps://doi.org/10.1108/srj-11-2018-0298](ttps://doi.org/10.1108/srj-11-2018-0298%20)

Norton, T. A., Zacher, H., & Ashkanasy, N. M. (2014). Organisational sustainability policies and employee green Behavior: The mediating role of work climate perceptions. *Journal of Environmental Psychology*, *38*, 49-54. h[ttps://doi.org/https://doi.org/10.1016/j.jenvp.2013.12.008](ttps://doi.org/https://doi.org/10.1016/j.jenvp.2013.12.008%20)

Norton, T. A., Zacher, H., Parker, S. L., & Ashkanasy, N. M. (2017). Bridging the gap between green behavioral intentions and employee green behavior: The role of green psychological climate. *Journal of Organizational Behavior*, *38*(7), 996-1015. h[ttps://doi.org/https://doi.org/10.1002/job.2178](ttps://doi.org/https://doi.org/10.1002/job.2178%20)

Okumus, F., Köseoglu, M. A., Chan, E., Hon, A., & Avci, U. (2019). How do hotel employees' environmental attitudes and intentions to implement green practices relate to their ecological behavior? *Journal of Hospitality and Tourism Management*, *39*, 193-200. h[ttps://doi.org/10.1016/j.jhtm.2019.04.008](ttps://doi.org/10.1016/j.jhtm.2019.04.008%20)

Paillé, P., & Boiral, O. (2013). Pro-environmental behavior at work: Construct validity and determinants. *Journal of Environmental Psychology*, *36*, 118-128. h[ttps://doi.org/10.1016/j.jenvp.2013.07.014](ttps://doi.org/10.1016/j.jenvp.2013.07.014%20)

Paillé, P., Boiral, O., & Chen, Y. (2013). Linking environmental management practices and organizational citizenship Behavior for the environment: A social exchange perspective. *The International Journal of Human Resource Management*, *24*(18), 3552-3575. h[ttps://doi.org/10.1080/09585192.2013.777934](ttps://doi.org/10.1080/09585192.2013.777934%20)

Paillé, P., Chen, Y., Boiral, O., & Jin, J. (2014). The Impact of Human Resource Management on Environmental Performance: An Employee-Level Study. *Journal of Business Ethics*, *121*(3), 451-466. h[ttps://doi.org/10.1007/s10551-013-1732-0](ttps://doi.org/10.1007/s10551-013-1732-0%20)

Paillé, P., & Mejía-Morelos, J. H. (2014). Antecedents of pro-environmental Behaviors at work: The moderating influence of psychological contract breach. *Journal of Environmental Psychology*, *38*, 124-131. h[ttps://doi.org/https://doi.org/10.1016/j.jenvp.2014.01.004](ttps://doi.org/https://doi.org/10.1016/j.jenvp.2014.01.004%20)

Paillé, P., Mejía-Morelos, J. H., Marché-Paillé, A., Chen, C. C., & Chen, Y. (2016). Corporate Greening, Exchange Process Among Co-workers, and Ethics of Care: An Empirical Study on the Determinants of Pro-environmental Behaviors at Coworkers-Level. *Journal of Business Ethics*, *136*(3), 655-673. h[ttps://doi.org/10.1007/s10551-015-2537-0](ttps://doi.org/10.1007/s10551-015-2537-0%20)

Paillé, P., Mejía Morelos, J. H., Raineri, N., & Stinglhamber, F. (2019). The influence of the immediate manager on the avoidance of non-green behaviors in the workplace: A three-wave moderated-mediation model. *Journal of Business Ethics*, *155*(3), 723-740. h[ttps://doi.org/10.1007/s10551-017-3519-1](ttps://doi.org/10.1007/s10551-017-3519-1%20)

Paillé, P., & Morelos, J. H. M. (2017). Modelling how managers support their subordinates toward environmental sustainability: A moderated-mediation study. *Journal of Applied Business Research*, *33*(4), 721-730.

Paillé, P., Raineri, N., & Boiral, O. (2019). Environmental behavior on and off the job: A configurational approach. *Journal of Business Ethics*, *158*(1), 253-268. h[ttps://doi.org/10.1007/s10551-017-3758-1](ttps://doi.org/10.1007/s10551-017-3758-1%20)

Pandur, V., & Albulescu, P. (2016). How to promote pro-environmental Behavior in organizations: the message matters. *Psihologia Resurselor Umane*, *14*(1), 69-82.

Pellegrini, C., Rizzi, F., & Frey, M. (2018). The role of sustainable human resource practices in influencing employee behavior for corporate sustainability. *Business Strategy and the Environment*, *27*(8), 1221-1232. h[ttps://doi.org/10.1002/bse.2064](ttps://doi.org/10.1002/bse.2064%20)

Peng, X., & Lee, S. (2019). Self-discipline or self-interest? The antecedents of hotel employees' pro-environmental Behaviors. *Journal of Sustainable Tourism*, *27*(9), 1457-1476. h[ttps://doi.org/10.1080/09669582.2019.1632320](ttps://doi.org/10.1080/09669582.2019.1632320%20)

Pham, N. T., Phan, Q. P. T., Tučková, Z., Vo, N., & Nguyen, L. H. L. (2018). Enhancing the organizational citizenship behavior for the environment: the roles of green training and organizational culture. *Management & Marketing*, *13*(4), 1174-1189. h[ttps://doi.org/10.2478/mmcks-2018-0030](ttps://doi.org/10.2478/mmcks-2018-0030%20)

Pham, N. T., Tučková, Z., & Chiappetta Jabbour, C. J. (2019). Greening the hospitality industry: How do green human resource management practices influence organizational citizenship behavior in hotels? A mixed-methods study. *Tourism Management*, *72*, 386-399. h[ttps://doi.org/10.1016/j.tourman.2018.12.008](ttps://doi.org/10.1016/j.tourman.2018.12.008%20)

Raineri, N., & Paillé, P. (2016). Linking corporate policy and supervisory support with environmental citizenship behaviors: The role of employee environmental beliefs and commitment. *Journal of Business Ethics*, *137*(1), 129-148. h[ttps://doi.org/10.1007/s10551-015-2548-x](ttps://doi.org/10.1007/s10551-015-2548-x%20)

Rayner, J., & Morgan, D. (2018). An empirical study of ‘green’ workplace Behaviors: ability, motivation and opportunity. *Asia Pacific Journal of Human Resources*, *56*(1), 56-78. h[ttps://doi.org/10.1111/1744-7941.12151](ttps://doi.org/10.1111/1744-7941.12151%20)

Rezapouraghdam, H., Alipour, H., & Darvishmotevali, M. (2018). Employee workplace spirituality and pro-environmental behavior in the hotel industry. *Journal of Sustainable Tourism*, *26*(5), 740-758. h[ttps://doi.org/10.1080/09669582.2017.1409229](ttps://doi.org/10.1080/09669582.2017.1409229%20)

Robertson, J. L., & Barling, J. (2013). Greening organizations through leaders' influence on employees' pro-environmental behaviors. *Journal of Organizational Behavior*, *34*(2), 176-194. h[ttps://doi.org/https://doi.org/10.1002/job.1820](ttps://doi.org/https://doi.org/10.1002/job.1820%20)

Robertson, J. L., & Carleton, E. (2018). Uncovering How and When Environmental Leadership Affects Employees’ Voluntary Pro-environmental Behavior. *Journal of Leadership & Organizational Studies*, *25*(2), 197-210. h[ttps://doi.org/10.1177/1548051817738940](ttps://doi.org/10.1177/1548051817738940%20)

Ruepert, A. M., Keizer, K., & Steg, L. (2017). The relationship between Corporate Environmental Responsibility, employees’ biospheric values and pro-environmental Behavior at work. *Journal of Environmental Psychology*, *54*, 65-78. h[ttps://doi.org/10.1016/j.jenvp.2017.10.006](ttps://doi.org/10.1016/j.jenvp.2017.10.006%20)

Saeed, B. B., Afsar, B., Hafeez, S., Khan, I., Tahir, M., & Afridi, M. A. (2019). Promoting employee's proenvironmental behavior through green human resource management practices. *Corporate Social Responsibility and Environmental Management*, *26*(2), 424-438. h[ttps://doi.org/10.1002/csr.1694](ttps://doi.org/10.1002/csr.1694%20)

Safari, A., Salehzadeh, R., Panahi, R., & Abolghasemian, S. (2018). Multiple pathways linking environmental knowledge and awareness to employees’ green behavior. *Corporate Governance: The International Journal of Business in Society*, *18*(1), 81-103. h[ttps://doi.org/10.1108/cg-08-2016-0168](ttps://doi.org/10.1108/cg-08-2016-0168%20)

Saifulina, N., & Carballo-Penela, A. (2017). Promoting Sustainable Development at an Organizational Level: An Analysis of the Drivers of Workplace Environmentally Friendly Behavior of Employees. *Sustainable Development*, *25*(4), 299-310. h[ttps://doi.org/10.1002/sd.1654](ttps://doi.org/10.1002/sd.1654%20)

Stritch, J. M., & Christensen, R. K. (2016). Going Green in Public Organizations. *The American Review of Public Administration*, *46*(3), 337-355. h[ttps://doi.org/10.1177/0275074014552470](ttps://doi.org/10.1177/0275074014552470%20)

Su, L., & Swanson, S. R. (2019). Perceived corporate social responsibility's impact on the well-being and supportive green behaviors of hotel employees: The mediating role of the employee-corporate relationship. *Tourism Management*, *72*, 437-450. h[ttps://doi.org/10.1016/j.tourman.2019.01.009](ttps://doi.org/10.1016/j.tourman.2019.01.009%20)

Subramanian, N., Abdulrahman, M. D., Wu, L., & Nath, P. (2016). Green competence framework: evidence from China. *The International Journal of Human Resource Management*, *27*(2), 151-172. h[ttps://doi.org/10.1080/09585192.2015.1047394](ttps://doi.org/10.1080/09585192.2015.1047394%20)

Tariq, M., Yasir, M., & Majid, A. (2020). Promoting employees' environmental performance in hospitality industry through environmental attitude and ecological behavior: Moderating role of managers' environmental commitment. *Corporate Social Responsibility and Environmental Management*, *27*(6), 3006-3017. h[ttps://doi.org/10.1002/csr.2019](ttps://doi.org/10.1002/csr.2019%20)

Temminck, E., Mearns, K., & Fruhen, L. (2015). Motivating employees towards sustainable Behavior. *Business Strategy and the Environment*, *24*(6), 402-412. h[ttps://doi.org/10.1002/bse.1827](ttps://doi.org/10.1002/bse.1827%20)

Teng, C.-C., Horng, J.-S., Hu, M.-L. M., & Chen, P.-C. (2014). Exploring the Energy and Carbon Literacy Structure for Hospitality and Tourism Practitioners: Evidence from Hotel Employees in Taiwan. *Asia Pacific Journal of Tourism Research*, *19*(4), 451-468. h[ttps://doi.org/10.1080/10941665.2013.764336](ttps://doi.org/10.1080/10941665.2013.764336%20)

Testa, F., Corsini, F., Gusmerotti, N. M., & Iraldo, F. (2020). Predictors of organizational citizenship behavior in relation to environmental and health & safety issues. *The International Journal of Human Resource Management*, *31*(13), 1705-1738. h[ttps://doi.org/10.1080/09585192.2017.1423099](ttps://doi.org/10.1080/09585192.2017.1423099%20)

Tian, H., Zhang, J., & Li, J. (2020). The relationship between pro-environmental attitude and employee green behavior: the role of motivational states and green work climate perceptions. *Environmental Science and Pollution Research*, *27*(7), 7341-7352. h[ttps://doi.org/10.1007/s11356-019-07393-z](ttps://doi.org/10.1007/s11356-019-07393-z%20)

Tian, Q., & Robertson, J. L. (2019). How and when does perceived CSR affect employees’ engagement in voluntary pro-environmental behavior? *Journal of Business Ethics*, *155*(2), 399-412. h[ttps://doi.org/10.1007/s10551-017-3497-3](ttps://doi.org/10.1007/s10551-017-3497-3%20)

Tosti-Kharas, J., Lamm, E., & Thomas, T. E. (2017). Organization OR environment? Disentangling employees’ rationales behind organizational citizenship behavior for the environment. *Organization & Environment*, *30*(3), 187-210. h[ttps://doi.org/10.1177/1086026616668381](ttps://doi.org/10.1177/1086026616668381%20)

Tudor, T. L., Barr, S. W., & Gilg, A. W. (2007). Strategies for improving recycling Behavior within the Cornwall National Health Service (NHS) in the UK. *Waste Manag Res*, *25*(6), 510-516. h[ttps://doi.org/10.1177/0734242X07082030](ttps://doi.org/10.1177/0734242X07082030%20)

Tudor, T. L., Barr, S. W., & Gilg, A. W. (2008). A novel conceptual framework for examining environmental behavior in large organizations: A case study of the Cornwall National Health Service (NHS) in the United Kingdom. *Environment and Behavior*, *40*(3), 426-450. h[ttps://doi.org/10.1177/0013916507300664](ttps://doi.org/10.1177/0013916507300664%20)

Verplanken, B., Walker, I., Davis, A., & Jurasek, M. (2008). Context change and travel mode choice: Combining the habit discontinuity and self-activation hypotheses. *Journal of Environmental Psychology*, *28*(2), 121-127. h[ttps://doi.org/10.1016/j.jenvp.2007.10.005](ttps://doi.org/10.1016/j.jenvp.2007.10.005%20)

Wahab, M. A. (2017). Relationships between religious work values, sustainable work Behaviors and sustainable energy consumptions. *Management Decision*, *55*(9), 1854-1867. h[ttps://doi.org/10.1108/md-01-2017-0039](ttps://doi.org/10.1108/md-01-2017-0039%20)

Wang, X., Zhou, K., & Liu, W. (2018). Value Congruence: A Study of Green Transformational Leadership and Employee Green Behavior. *Frontiers in psychology*, *9*, 1946. h[ttps://doi.org/10.3389/fpsyg.2018.01946](ttps://doi.org/10.3389/fpsyg.2018.01946%20)

Wang, Y.-F. (2016). Modeling predictors of restaurant employees’ green behavior: Comparison of six attitude-behavior models. *International Journal of Hospitality Management*, *58*, 66-81. h[ttps://doi.org/10.1016/j.ijhm.2016.07.007](ttps://doi.org/10.1016/j.ijhm.2016.07.007%20)

Zhao, H., Zhou, Q., He, P., & Jiang, C. (2019). How and when does socially responsible HRM affect employees’ organizational citizenship behaviors toward the environment? *Journal of Business Ethics*. h[ttps://doi.org/10.1007/s10551-019-04285-7](ttps://doi.org/10.1007/s10551-019-04285-7%20)

Zientara, P., & Zamojska, A. (2018). Green organizational climates and employee pro-environmental Behavior in the hotel industry. *Journal of Sustainable Tourism*, *26*(7), 1142-1159. <https://doi.org/10.1080/09669582.2016.1206554>

1. Note that two articles (Erdogan et al., 2015; Paillé et al., 2013) have respectively investigated two different forms of support, which explains the discrepancy between the number 21 and the 19 listed references [↑](#footnote-ref-1)
